# NifFinder: improved Nif protein prediction using SWeeP vectors and neural networks

**DOI:** 10.1093/bioadv/vbaf260

**Published:** 2025-10-16

**Authors:** Bruno Thiago de Lima Nichio, Roxana Beatriz Ribeiro Chaves, Jeroniza Nunes Marchaukoski, Fabio de Oliveira Pedrosa, Roberto Tadeu Raittz

**Affiliations:** Laboratory of Artificial Intelligence Applied to Bioinformatics (AIBIA), Professional and Technical Education Sector (SEPT) - UFPR, Curitiba, Paraná, 81520-260, Brazil; Department of Biochemistry, Biological Sciences Sector – Federal University of Paraná (UFPR), Curitiba, Paraná, 81531-980, Brazil; Department of Biochemistry, Biological Sciences Sector – Federal University of Paraná (UFPR), Curitiba, Paraná, 81531-980, Brazil; Laboratory of Artificial Intelligence Applied to Bioinformatics (AIBIA), Professional and Technical Education Sector (SEPT) - UFPR, Curitiba, Paraná, 81520-260, Brazil; Laboratory of Artificial Intelligence Applied to Bioinformatics (AIBIA), Professional and Technical Education Sector (SEPT) - UFPR, Curitiba, Paraná, 81520-260, Brazil; Department of Biochemistry, Biological Sciences Sector – Federal University of Paraná (UFPR), Curitiba, Paraná, 81531-980, Brazil; Laboratory of Artificial Intelligence Applied to Bioinformatics (AIBIA), Professional and Technical Education Sector (SEPT) - UFPR, Curitiba, Paraná, 81520-260, Brazil; Department of Biochemistry, Biological Sciences Sector – Federal University of Paraná (UFPR), Curitiba, Paraná, 81531-980, Brazil

## Abstract

**Motivation:**

Biological nitrogen fixation is a vital process for global ecosystems and agriculture; however, the diversity and complexity of *nif* genes present significant challenges for the accurate identification of Nif proteins. Existing computational tools are often limited to a narrow subset of *nif* genes, leaving many important protein classes unexplored. NifFinder was developed to address this gap, combining SWeeP vector representation with neural network models to predict up to 24 different Nif proteins. By expanding the predictive scope and improving accuracy, NifFinder provides a more comprehensive and reliable framework to study nitrogen fixation, supporting both evolutionary insights and applications in agricultural sustainability.

**Results:**

We present NifFinder, a computational framework that integrates SWeeP vector encoding with neural network classifiers to predict up to 24 different Nif protein classes across Archaea and Bacteria. NifFinder achieved an average accuracy of 84.31%, with sensitivity (86.49%), precision (81.97%), F1-score (82.33%), and a class correlation coefficient of 0.94. Benchmarking against Nif curated resources showed strong agreement and robust classification even under class imbalance. By expanding beyond traditional subsets of *nif* genes, NifFinder enables more reliable genome-wide identification of Nif proteins.

**Availability and implementation:**

The NifFinder installation instructions and source code can be accessed at https://sourceforge.net/projects/NifFinder.

## 1 Introduction

### 1.1 Comprehensive coverage of Nif proteins: a current challenge

Biological nitrogen fixation (BNF) is a fundamental process carried out by diazotrophic organisms, in which atmospheric nitrogen (N_2_) is converted into ammonia, providing bioavailable nitrogen to sustain ecosystems and agricultural productivity (Fowler *et al.* 2013, Vitousek *et al.* 2013). This transformation is catalyzed by nitrogenase enzymes, with the molybdenum-dependent nitrogenase (Mo-nitrogenase) being the most widespread and efficient form (Burgess and Lowe 1996). The Mo-nitrogenase system is encoded by a complex set of up to 24 *nif* genes, which together produce the structural subunits and accessory proteins required for enzyme assembly, regulation, and function (Raymond and Blankenship 2004, Rubio and Ludden 2008, Dos Santos *et al.* 2012). The diversity of *nif* genes across diazotrophic taxa has been extensively documented, reflecting both evolutionary innovation and ecological adaptation (Boyd and Peters 2013, Gaby and Buckley 2014). However, this diversity also presents persistent challenges for computational prediction and classification. Distinguishing among 24 different Nif proteins is particularly difficult in less-characterized or novel organisms, where annotations may be incomplete or ambiguous. Moreover, accessory Nif proteins, though critical for nitrogenase assembly and activity, remain underrepresented in existing prediction frameworks, which often focus narrowly on core subunits such as *nifH*, *nifD*, and *nifK* (Zehr and Turner 2001, Raymond *et al.* 2004, Boyd *et al.* 2015). Despite decades of research, the evolutionary complexity and functional diversity of *nif* genes continue to challenge accurate classification. There is thus a pressing need for comprehensive and robust computational approaches capable of identifying the full spectrum of Nif proteins across diverse microbial genomes.

The accurate identification of *nif* genes and their products not only deepens our understanding of the evolutionary mechanisms underlying biological nitrogen fixation but also enhances the prospects for their applied utilization. For example, the ability to reliably detect Nif proteins within genomes and metagenomes provides a critical framework for assessing the potential of previously uncharacterized microbial lineages to contribute to biological nitrogen fixation (Delmont *et al.* 2018). This has direct implications for agricultural sustainability, where harnessing or enhancing microbial nitrogen fixation could reduce dependence on synthetic fertilizers and contribute to more sustainable crop production (Beatty and Good 2011, Mus *et al.* 2016).

### 1.2 Lack of computational tools developed for *nif* genes or *nif* gene products prediction

Computational tools have been developed to address the need for reliable prediction of *nif* genes or Nif proteins encoded by *nif* genes. The NifH MiSeq Illumina amplicon Analysis Pipeline (NifMAP) and the NifH protein database are prominent examples (Gaby and Buckley 2014, Angel *et al.* 2018). NifMAP utilizes Hidden Markov Models (HMM) to filter out homologous genes to the nifH gene, a critical marker for diazotrophs in metagenomic datasets. The *nifH* gene, a component of the nitrogenase complex, is widely utilized to assess the presence and diversity of nitrogen-fixing organisms in environmental samples. The NifH sequence database offers a comprehensive collection of *nifH* sequences from diverse organisms, enabling comparative studies and identifying potential diazotrophs in genomic and metagenomic datasets. Additionally, NFixDB serves as a curated database for nitrogen-fixing organisms and genes, offering a comprehensive (over 4000 *nifHDK* genes) and integrated whole-genome database for diazotrophs, encompassing all nitrogenases (*nifHDK*, *vnfHDK*, *anfHDK*) and nitrogenase-like enzymes linked to ribosomal RNA operons (Bellanger *et al.* 2024).

Despite the utility of these tools, gaps remain in the field. Specifically, many existing tools are limited to identifying the *nifH* gene or *nifHDK* clusters and do not account for the full spectrum of *nif* genes or their diverse configurations across different species. NifPRED introduced a pioneering approach for the computational identification of Nif proteins encoded by six key categories of *nif* genes (*nifH*, *nifD*, *nifK*, *nifE*, *nifN*, and *nifB*). The algorithm uses the composition-transition-distribution (CTD) feature to convert protein sequences into numerical feature vectors. For prediction, it utilizes a Support Vector Machine (SVM) with a Radial Basis Function (RBF) Neural Network kernel, configured with default parameters, ensuring effective classification of Nif proteins (with an overall accuracy of more than 90%) (Meher *et al.* 2018). However, there are at least 18 *nif* genes for which no mapping strategies have been established. This is primarily due to the extensive diversity and complexity inherent to each type of nif gene and its Nif protein sequences (Dixon and Kahn 2004, Raymond *et al.* 2004, Dos Santos *et al.* 2012).

In summary, traditional computational approaches for identifying Nif proteins often focus on a restricted set of core genes (e.g. *nifH*, *nifD*, *nifK*), which limits their ability to capture the broader functional landscape. With the advent of large-scale genome sequencing, there is a pressing need for comprehensive, high-accuracy predictors that can identify diverse *nif*-associated proteins across taxonomic groups.

### 1.3 NifFinder: combining vectorial representations of sequences with neural networks

Here, we introduce NifFinder as a tool designed to predict up to 24 Nif proteins [20 Nif proteins described in *Klebsiella quasivariicola* 342 (Arnold *et al.* 1988) plus NifP, NifO, NifI1I2, and NifXa] and bifunctional proteins with Mo-nitrogenase activity (NifEN, NifNB). By integrating Spaced Words Projection (SWeeP) vectors (De Pierri *et al.* 2020) with advanced artificial neural network architectures, including the Radial Basis Function (RBF) Neural Network and the Multi-Layer Perceptron (MLP), NifFinder provides a robust framework for the accurate classification of Nif proteins. The development of NifFinder represents a significant step forward in the field, offering a more comprehensive approach to predicting and classifying Nif proteins encoded by *nif* genes. Our goal is to expand the predictive scope of Nif protein identification, improve accuracy, and provide a robust framework for exploring nitrogenase diversity in microbial genomes. Therefore, NifFinder provides a valuable resource for researchers studying nitrogen fixation and the evolutionary dynamics of diazotrophic organisms.

## 2 Methods

### 2.1 Data collection and preprocessing

Annotated Nif protein sequences were curated from a reference of 662 whole genomes in both Bacteria and Archaea, covering canonical nitrogenase subunits and accessory proteins (Nichio *et al.* 2025) and downloaded at NCBI File Transfer Protocol (FTP) (https://ftp.ncbi.nlm.nih.gov/genomes/). The coding sequences (.faa extension) of these whole genomes were stratified by class to minimize redundancy utilizing RAFTS³G (https://sourceforge.net/projects/rafts-g/) configured with a 0.5 similarity threshold between sequences, using the binary search function.

We used the SWeeP method to transform amino acid sequences into fixed-length numerical vectors (De Pierri *et al.* 2020). The SWeeP properties permit the use of vectors preserving inter-sequence comparability. The protein sequences used for the Neural network model training were vectorized using the rSWeeP version, available at Bioconductor (Fernandes 2020). The parameters for SWeeP included spaced words “11011” and a projection matrix (W) of 1369 (37 × 37) coordinates.

### 2.2 Neural network models training

In summary, each Nif class was represented in SWeeP vectors and independently trained as a true dataset for neural networks. The false datasets comprised sequences from random clusters obtained by RAFTS³G clustering, with a sample ratio of approximately 1:2 (one Nif sequence for two non-Nif sequences). The models were based on Radial Basis Function (RBF) and Multi-Layer Perceptron (MLP) learning models. The dataset was partitioned into training (70%) and testing (30%) sets, with stratified sampling to address class imbalance. Hyperparameters were optimized through cross-validation, with the MLP configured with a 5:3:1 layer structure and the RBF set to a basis function size of 51. The optimal model was selected considering the sensitivity, accuracy, and F1-score balance. Among the models, the networks with the highest F1 score were selected, and in cases where metrics matched, the models with the highest accuracy were chosen.

### 2.3 Evaluation metrics and datasets

Performance was assessed using accuracy, sensitivity, precision, F1-score, and class correlation coefficients. The predictive performance of Nif sequences in whole genomes was performed using the 662 microbial genomes described by Nichio *et al.* (2025). Additionally, five whole genomes of Archaea and Bacteria were selected to compare the prediction with NifPred: *Methanobacterium congolense* isolate Buetzberg (MCBB) (Kessler *et al.* 1998), *Paenibacillus borealis* DSM 13188 (ASM75866v1) (Xie *et al.* 2014), *Aphanizomenon flos-aquae* DEX188 (ASM1734685v1) (Thiel 2019), *Klebsiella variicola* 342 (ASM1956v1) (Arnold *et al.* 1988), and *Azospirillum brasilense* strain Sp7 (ASM131501v1) (Zhang *et al.* 1997). Finally, we compare the NFixDB catalogue (release 3.1.0) from the Zenodo repository (https://zenodo.org/records/10975171) to analyze the generalization capacity of NifFinder. Additional benchmarking included confusion matrices and per-class performance breakdown. The scripts, datasets, and trained models are available in the Supplementary material.

## 3 Results

### 3.1 NifFinder application strategy

**Figure 1.**
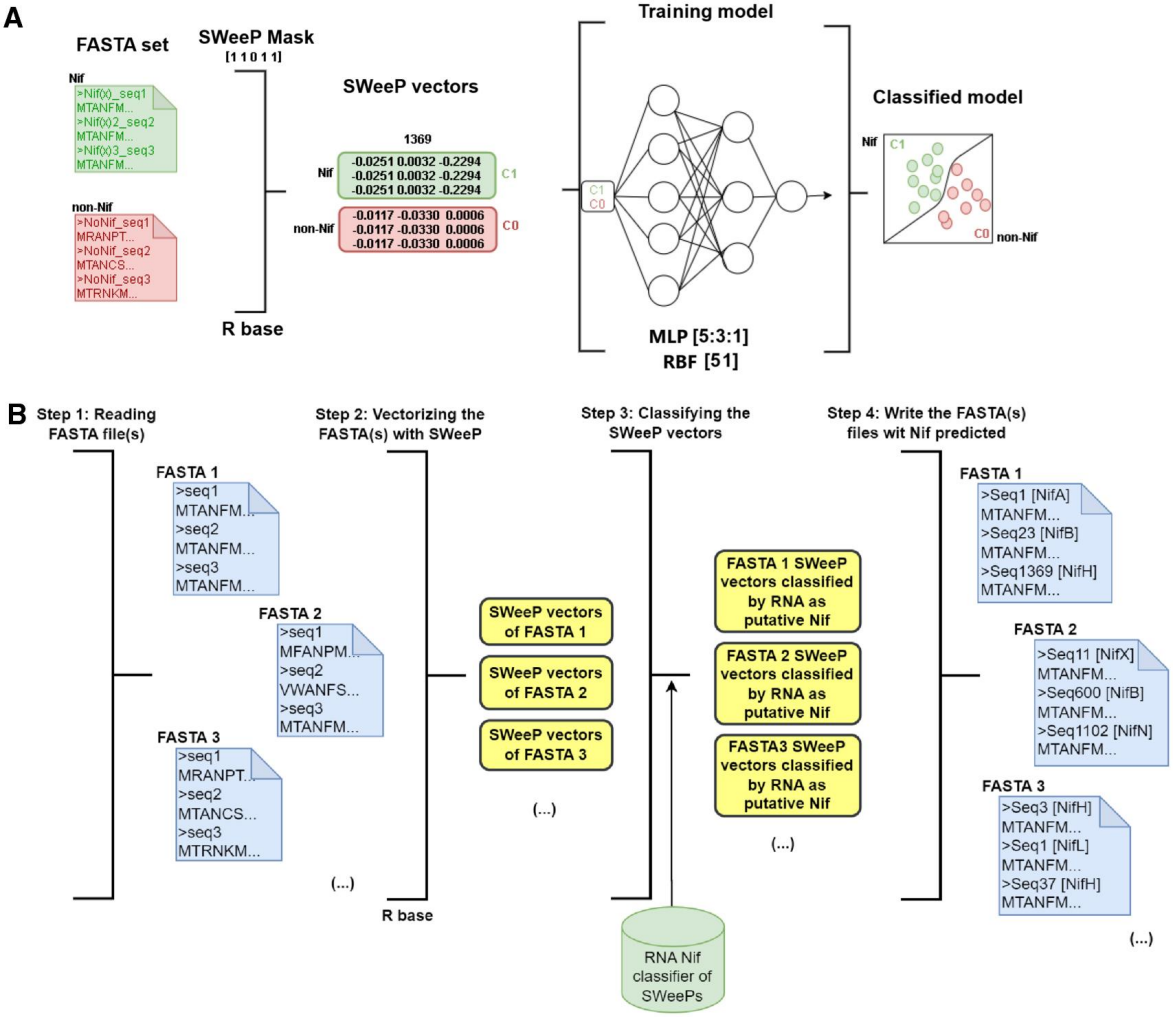
(A) The NifFinder model training dataset using the SWeeP method. The Nif (Class1—“true”) and non-Nif (Class0—“false”) sequences were projected in SWeeP vectors (using Mask “11011” and a 1369-coordinate projection for vector representation). These vectors were trained and tested using the models MLP (layers 5:3:1) and RBF (radial basis function value equal to 51). (B) NifFinder overall workflow process. The application strategy of NifFinder involves predicting Nif proteins across one or various amino acid sequences (until sequences are extracted from whole genomes) in FASTA format. The NifFinder algorithm operates in four main steps. First, each input FASTA file (amino acid sequences) is converted into its respective SWeeP vector (Steps 1 and 2). Then, these vectors are scanned to identify potential Nif candidates by comparing them with a Nif classifier for vectorized sequences, selecting likely Nif proteins (Step 3). Finally, NifFinder labeled output files with each predicted Nif protein.

The Nif network model development strategy in NifFinder for a single Nif class (e.g. for creating a predictor network for NifH) is illustrated in Fig. 1A. The NifFinder application strategy (Fig. 1B) first converts each input FASTA file (amino acid sequences) into its corresponding SWeeP vector (Steps 1 and 2). NifFinder then scans these vectors to identify potential Nif candidates by comparing them with a Nif classifier for vectorized sequences, selecting putative Nif proteins (Step 3). Finally, NifFinder outputs two files: a matrix structure (.mat) compatible with MATLAB^®^ for further manipulation and the FASTA file with predicted Nif proteins, each labeled with its corresponding Nif type in the header. NifFinder was compiled using MATLAB^®^ Compiler Runtime (MCR) v8.0.

### 3.2 Overall predictive performance in whole genomes: strong agreement between NifFinder predictions and curated annotations

The development of Artificial Neuronal Networks (ANNs) in NifFinder demonstrated satisfactory and robust performance, as evidenced by the confusion matrix analyses during classification validation (average F1 score = 0.952), using both MLP and RBF neural networks (Table 1, available as supplementary data at *Bioinformatics Advances* online). The performance across both models was consistent, with only slight differences in their best F1 scores. Overall, the models exhibit robust classification accuracy, indicating effective identification of Nif proteins.

NifFinder’s predictive performance was assessed using 662 diazotrophic whole genomes. Its predictions (Table 3, available as supplementary data at *Bioinformatics Advances* online) were benchmarked against the previously curated annotated Nif protein sequences described in Nichio *et al.* (2025) and provided in the same dataset (Table 2, available as supplementary data at *Bioinformatics Advances* online). Overall, the tool achieved an average accuracy of 84.31%, demonstrating strong agreement between predicted and annotated Nif protein types across both Archaea and Bacteria domains. Beyond accuracy, the evaluation also highlighted an average sensitivity of 86.49%, precision of 81.97%, and F1-score of 82.33%, reinforcing the robustness of NifFinder even in the context of class imbalance. A detailed breakdown of these metrics is provided in Table 4, available as supplementary data at *Bioinformatics Advances* online. Finally, the coefficient of the relation of classes identified with NifFinder to Nif annotations was 0.94, indicating strong agreement with curated annotations.

Despite uneven class distributions, NifFinder maintained stable predictive power, with sensitivity and precision consistently above 80%. Minority classes, though more variable, remained identifiable within acceptable statistical confidence.

### 3.3 Beyond core genes: NifFinder accurately captures the full spectrum of Nif proteins

We evaluated the performance of NifFinder and NifPRED in classifying Nif proteins using datasets derived from *nif* gene clusters of five reference organisms representing Archaea and Bacteria (*Methanobacterium congolense* isolate Buetzberg, *Paenibacillus borealis* DSM 13188, *Aphanizomenon flos-aquae* DEX188, *K. variicola* 342, and *Azospirillum brasilense* strain Sp7) (Table 5, available as supplementary data at *Bioinformatics Advances* online). The dataset included 71 confirmed Nif proteins and 39 non-Nif proteins.

NifPRED accurately identified 28 out of 29 (96.4%), considering only NifHDKENB proteins. However, it misclassified nine sequences among other Nif types, such as incorrectly assigning NifN as NifK in M. congolense or NifB as NifU in *K. variicola*. Additionally, NifPRED incorrectly labeled 36 Nif proteins as “non-Nif,” while successfully classifying 40 non-Nif protein sequences.

In contrast, NifFinder successfully classified all NifHDKENB proteins (29/29) and accurately identified 74 Nif protein types, including NifHDKENB, within the dataset. Importantly, as NifFinder is specifically designed to predict only Nif sequences, any sequence not classified as a Nif protein is automatically categorized as non-Nif. This approach resulted in 39 sequences being correctly identified as non-Nif, ensuring a clear distinction between Nif and non-Nif sequences and reinforcing the tool’s precision in handling diverse datasets.

Although a direct accuracy comparison is complex, the explicit advantage in this context lies in NifFinder’s broader scope, particularly its ability to identify accessory proteins that are overlooked by other tools. Unlike previous predictors restricted to a subset of *nif* genes, NifFinder successfully identified accessory proteins and auxiliary components critical for nitrogenase assembly and function. This expanded coverage provides a more holistic perspective of *nif* operon diversity.

### 3.4 Validating the generalization performance of NifFinder through the curated Nif database

We utilized NFixDB to validate the performance of the generalization of NifFinder. NFixDB contains 4085, 4162, and 4158 sequences for NifH, NifD, and NifK, respectively, in addition to other non-Nif sequences (i.e. alternative nitrogenases and sequences related to light-independent reduction of protochlorophyllide). We found that NifFinder correctly classified 4059 sequences (99.3%) for NifH, 4020 (96.6%) for NifD, and 3919 (94.2%) for NifK (Table 6, available as supplementary data at *Bioinformatics Advances* online).

Through a deeper analysis of the sequences that showed discrepancies with the NifD annotation in NFixDB, we found that all 43 sequences (100.0%) predicted as NifE by NifFinder exhibit strong similarity to NifE sequences from the RefSeq database, as well as to the NifEN sequence. Similarly, among the NifK sequences in NFixDB that NifFinder classified as NifN or NifNB, we observed that 37 out of 180 sequences (20.5%) predicted as NifN show strong similarity to NifE sequences from RefSeq, as well as to the bifunctional NifNB protein. A detailed breakdown of these metrics is provided in Table 7, available as supplementary data at *Bioinformatics Advances* online.

The discrepancies arise from the methodological differences between NFixDB, which relies on HMM-based curation, and NifFinder, which was designed using artificial neural networks (ANNs). HMMs detect conserved sequence motifs through alignment-based profiles, whereas ANNs capture nonlinear patterns from vectorized representations, allowing greater flexibility but also leading to occasional divergences in classification. These complementary approaches naturally result in some discrepancies between the two tools.

## 4 Conclusion

NifFinder marks a significant advance in the computational prediction of Mo-nitrogenase proteins. By uniquely integrating SWeeP vector representation with neural networks, our tool expands the predictive scope to 24 distinct Nif classes, moving far beyond the core subunits targeted by existing methods. We demonstrated that NifFinder robustly identifies not only canonical nitrogenase components but also the critical spectrum of accessory proteins, a capability validated across hundreds of genomes and against the curated NFixDB database.

This expanded coverage provides a more holistic and reliable framework for mining genomic data, making NifFinder an invaluable asset for evolutionary biology, microbial ecology, and agricultural biotechnology. Future work will focus on incorporating alternative nitrogenases, extending the analysis to metagenomic data, and deploying a public web server to broaden the tool’s accessibility and impact on nitrogen fixation research.

## Supplementary Material

vbaf260_Supplementary_Data

## Data Availability

The NifFinder datasets and trained models are available in the supplementary content and at the link: https://sourceforge.net/projects/niffinder/.

## References

[vbaf260-B1] Angel R , Nepel M, Panhölzl C et al Evaluation of primers targeting the diazotroph functional gene and development of NifMAP—a bioinformatics pipeline for analyzing nifH amplicon data. Front Microbiol 2018;9:703. 29760683 10.3389/fmicb.2018.00703 PMC5936773

[vbaf260-B2] Arnold W , Rump A, Klipp W et al Nucleotide sequence of a 24,206-base-pair DNA fragment carrying the entire nitrogen fixation gene cluster of Klebsiella Pneumoniae. J Mol Biol 1988;203:715–38. 3062178 10.1016/0022-2836(88)90205-7

[vbaf260-B3] Beatty PH , Good AG. Plant science. Future prospects for cereals that fix nitrogen. Science (New York NY) 2011;333:416–7. 10.1126/science.1209467 21778391

[vbaf260-B4] Bellanger M , Figueroa JL III, Tiemann L et al NFixDB (nitrogen fixation DataBase)-a comprehensive integrated database for robust ’Omics analysis of diazotrophs. NAR Genom Bioinform 2024;6:lqae063. 38846350 10.1093/nargab/lqae063 PMC11155484

[vbaf260-B5] Boyd ES , Garcia Costas AM, Hamilton TL et al Evolution of molybdenum nitrogenase during the transition from anaerobic to aerobic metabolism. J Bacteriol 2015;197:1690–9. 25733617 10.1128/JB.02611-14 PMC4403663

[vbaf260-B6] Boyd ES , Peters JW. New insights into the evolutionary history of biological nitrogen fixation. Front Microbiol 2013;4:201. 23935594 10.3389/fmicb.2013.00201 PMC3733012

[vbaf260-B7] Burgess BK , Lowe DJ. Mechanism of molybdenum nitrogenase. Chem Rev 1996;96:2983–3012. 11848849 10.1021/cr950055x

[vbaf260-B8] De Pierri CR , Voyceik R, Santos de Mattos LGC et al SWeeP: representing large biological sequences datasets in compact vectors. Sci Rep 2020;10:91. 31919449 10.1038/s41598-019-55627-4 PMC6952362

[vbaf260-B9] Delmont TO , Quince C, Shaiber A et al Nitrogen-fixing populations of planctomycetes and proteobacteria are abundant in surface ocean metagenomes. Nat Microbiol 2018;3:804–13. 29891866 10.1038/s41564-018-0176-9 PMC6792437

[vbaf260-B10] Dixon R , Kahn D. Genetic regulation of biological nitrogen fixation. Nat Rev Microbiol 2004;2:621–31. 15263897 10.1038/nrmicro954

[vbaf260-B11] Dos Santos PC , Fang Z, Mason SW et al Distribution of nitrogen fixation and nitrogenase-like sequences amongst microbial genomes. BMC Genomics 2012;13:162. 22554235 10.1186/1471-2164-13-162 PMC3464626

[vbaf260-B12] Fernandes DR. rSWeeP. Bioconductor. 2020. 10.18129/B9.BIOC.RSWEEP.

[vbaf260-B13] Fowler D , Coyle M, Skiba U et al The global nitrogen cycle in the twenty-first century. Philos Trans R Soc Lond B Biol Sci 2013;368:20130164. 23713126 10.1098/rstb.2013.0164 PMC3682748

[vbaf260-B14] Gaby JC , Buckley DH. A comprehensive aligned nifH gene database: a multipurpose tool for studies of nitrogen-fixing bacteria. Database (Oxford) 2014;2014:bau001. 24501396 10.1093/database/bau001 PMC3915025

[vbaf260-B15] Kessler PS , Blank C, Leigh JA. The nif gene operon of the methanogenic archaeon Methanococcus Maripaludis. J Bacteriol 1998;180:1504–11. 9515920 10.1128/jb.180.6.1504-1511.1998 PMC107051

[vbaf260-B16] Meher PK , Sahu TK, Mohanty J et al nifPred: proteome-wide identification and categorization of nitrogen-fixation proteins of diaztrophs based on composition-transition-distribution features using support vector machine. Front Microbiol 2018;9:1100. 29896173 10.3389/fmicb.2018.01100 PMC5986947

[vbaf260-B17] Mus F , Crook MB, Garcia K et al Symbiotic nitrogen fixation and the challenges to its extension to nonlegumes. Appl Environ Microbiol 2016;82:3698–710. 27084023 10.1128/AEM.01055-16 PMC4907175

[vbaf260-B18] Nichio BTdL , Chaves RBR, Pedrosa FdO et al Exploring diazotrophic diversity: unveiling Nif core distribution and evolutionary patterns in Nitrogen-Fixing organisms. BMC Genomics 2025;26:81. 39871141 10.1186/s12864-024-10994-9 PMC11773926

[vbaf260-B19] Raymond J , Blankenship RE. Biosynthetic pathways, gene replacement and the antiquity of life. Geobiology 2004;2:199–203.

[vbaf260-B20] Raymond J , Siefert JL, Staples CR et al The natural history of nitrogen fixation. Mol Biol Evol 2004;21:541–54. 14694078 10.1093/molbev/msh047

[vbaf260-B21] Rubio LM , Ludden PW. Biosynthesis of the iron-molybdenum cofactor of nitrogenase. Annu Rev Microbiol 2008;62:93–111. 18429691 10.1146/annurev.micro.62.081307.162737

[vbaf260-B22] Thiel T. Organization and regulation of cyanobacterial nif gene clusters: implications for nitrogenase expression in plant cells. FEMS Microbiol Lett 2019;366:fnz077. 10.1093/femsle/fnz077 31062027

[vbaf260-B23] Vitousek PM , Menge DNL, Reed SC et al Biological nitrogen fixation: rates, patterns and ecological controls in terrestrial ecosystems. Philos Trans R Soc Lond B Biol Sci 2013;368:20130119. 23713117 10.1098/rstb.2013.0119 PMC3682739

[vbaf260-B24] Xie J-B , Du Z, Bai L et al Comparative genomic analysis of N2-fixing and Non-N2-fixing Paenibacillus Spp.: organization, evolution and expression of the nitrogen fixation genes. PLoS Genet 2014;10:e1004231. 24651173 10.1371/journal.pgen.1004231 PMC3961195

[vbaf260-B25] Zehr JP , Turner PJ. Nitrogen fixation: nitrogenase genes and gene expression. In: Moir JWB (ed.), *Methods in Microbiology*. San Diego, CA: Academic Press, 2001, 271–86. 10.1016/S0580-9517(01)30049-1 .

[vbaf260-B26] Zhang Y , Burris RH, Ludden PW et al Regulation of nitrogen fixation in *Azospirillum brasilense*. FEMS Microbiol Lett 1997;152:195–204. http://www.hpa.org.uk./infections/topics_az/antimicrobial_resistance/amr.pdf (7 January 2004, date last accessed). 9231412 10.1111/j.1574-6968.1997.tb10428.x

